# Brachytherapy for Soft Tissue Sarcoma: Maintaining Local Control While Minimizing Complications

**DOI:** 10.1002/jso.27999

**Published:** 2024-11-18

**Authors:** Julien Montreuil, Eric Kholodovsky, Moses Markowitz, Sergio Torralbas Fitz, Dominic Campano, Erik Geiger, Francis Hornicek, Brooke Crawford, Martin Keisch, H. Thomas Temple

**Affiliations:** ^1^ Department of Orthopaedics University of Miami Miller School of Medicine Miami Florida USA; ^2^ University of Miami Miller School of Medicine Miami Florida USA; ^3^ Cancer HealthCare Associates Miami Florida USA; ^4^ Department of Radiation Oncology University of Miami School of Medicine Miami Florida USA

**Keywords:** brachytherapy, soft tissue sarcoma, wound complications

## Abstract

**Background:**

This study aims to assess the clinical and oncologic outcomes of high‐dose brachytherapy (BRT) versus both preoperative and postoperative external beam radiation therapy (EBRT) in the setting of high‐grade soft tissue sarcoma.

**Methods:**

This is a retrospective cohort study of 144 patients treated surgically for soft tissue sarcoma at the same institution from 2010 to 2021. Patients treated for a soft tissue sarcoma with surgery and radiation therapy in the form of BRT, Neoadjuvant EBRT (Neo‐EBRT) or adjuvant EBRT (AD‐EBRT) were included.

**Results:**

56 patients were treated with BRT, 42 with Neo‐EBRT, and 46 with AD‐EBRT. There was a greater incidence of grouped wound complications in Neo‐EBRT with 50% compared to both BRT with 25% and AD‐EBRT with 28.3% (*p* = 0.02). Univariate and multivariate analysis showed that there was an increased risk of wound complications with Neo‐EBRT when compared to brachytherapy (*p* = 0.03 and *p* = 0.007, respectively). Univariate and multivariate analysis showed that there was no difference in risk of LR between treatment groups (*p* = 0.28).

**Conclusion:**

Brachytherapy is a valuable treatment modality that offers clinical and logistical advantages when compared to the conventional Neo‐EBRT in soft tissue sarcomas. Brachytherapy offers a lower risk of wound complications and a comparable local control. This manuscript presents decision‐making strategies for determining the appropriate radiation modality for specific circumstances.

## Summary

- Brachytherapy is a valuable treatment modality that offers clinical and logistical advantages when compared to the conventional Neo‐EBRT in soft tissue sarcomas.
- Brachytherapy offers a lower risk of wound complications and a comparable local control.
- This manuscript presents decision‐making strategies for determining the appropriate radiation modality for specific circumstances.

## 1 Introduction

Soft‐tissue sarcomas (STSs) are a heterogeneous and rare group of tumors comprising approximately 1% of neoplasms diagnosed in the adult population and account for over 20% of all pediatric solid malignant cancers [1]. Limb‐sparing surgery associated with radiotherapy (RT) seems to be the gold standard treatment for STSs, achieving local control rates of approximately 85%–90% and curative rates of 50% [2]. Brachytherapy (BRT) is an important radiotherapy modality for treating soft‐tissue sarcomas (STS) [1, 2]. It is used both as an adjunct to EBRT and as a monotherapy for these tumors [3]. BRT is a unique form of radiation therapy that allows for the precise placement of radioactive material directly into or near a tumor [4, 5]. This is performed either directly or through the use of catheters [4, 5]. This contrasts to external beam radiation therapy (EBRT), which is delivered using photons generated outside the patient [4]. The use of brachytherapy has now become a standard of care as both a monotherapy and as a boost to EBRT in some cancers such as cervical, prostate, and breast cancer [4]. In addition to the precision of brachytherapy, other advantages include a rapid dose falloff moving farther from the treatment site thus allowing for minimized radioactive exposure for distant tissue sites compared to conventional EBRT [4, 6]. This makes brachytherapy useful in recurrent soft tissue sarcomas even with prior radiation. Brachycatheters have also been used to manage unplanned excision of soft tissue sarcomas. Brachytherapy can be delivered over a shorter period than EBRT, which provides the patient with the clinical advantages of a shorter treatment period and lower indirect costs (travel and time from work), primarily when referred to a tertiary sarcoma center [3, 4, 7]. The disadvantages of brachytherapy are that it is technically demanding with regard to planning, catheter placement and delivery of the radiation source. [4, 8].

The efficacy and complication rates of brachytherapy in STS are still controversial. Multiple studies have concluded that surgical resection followed by high‐dose brachytherapy provides excellent local tumor control and non‐inferior complication rates for STSs [9, 10, 11, 12, 13, 14]. Other studies have concluded that brachytherapy treatment results in higher rates of perioperative wound complications and reoperation rates for infection control for STSs [15, 16, 17]. Data regarding these outcomes are sparse and require further investigation to provide a better understanding of treatment efficacy, which may ultimately help in optimizing patient selection and developing a more standardized approach for soft tissue sarcoma treatment.

**Figure 1:**
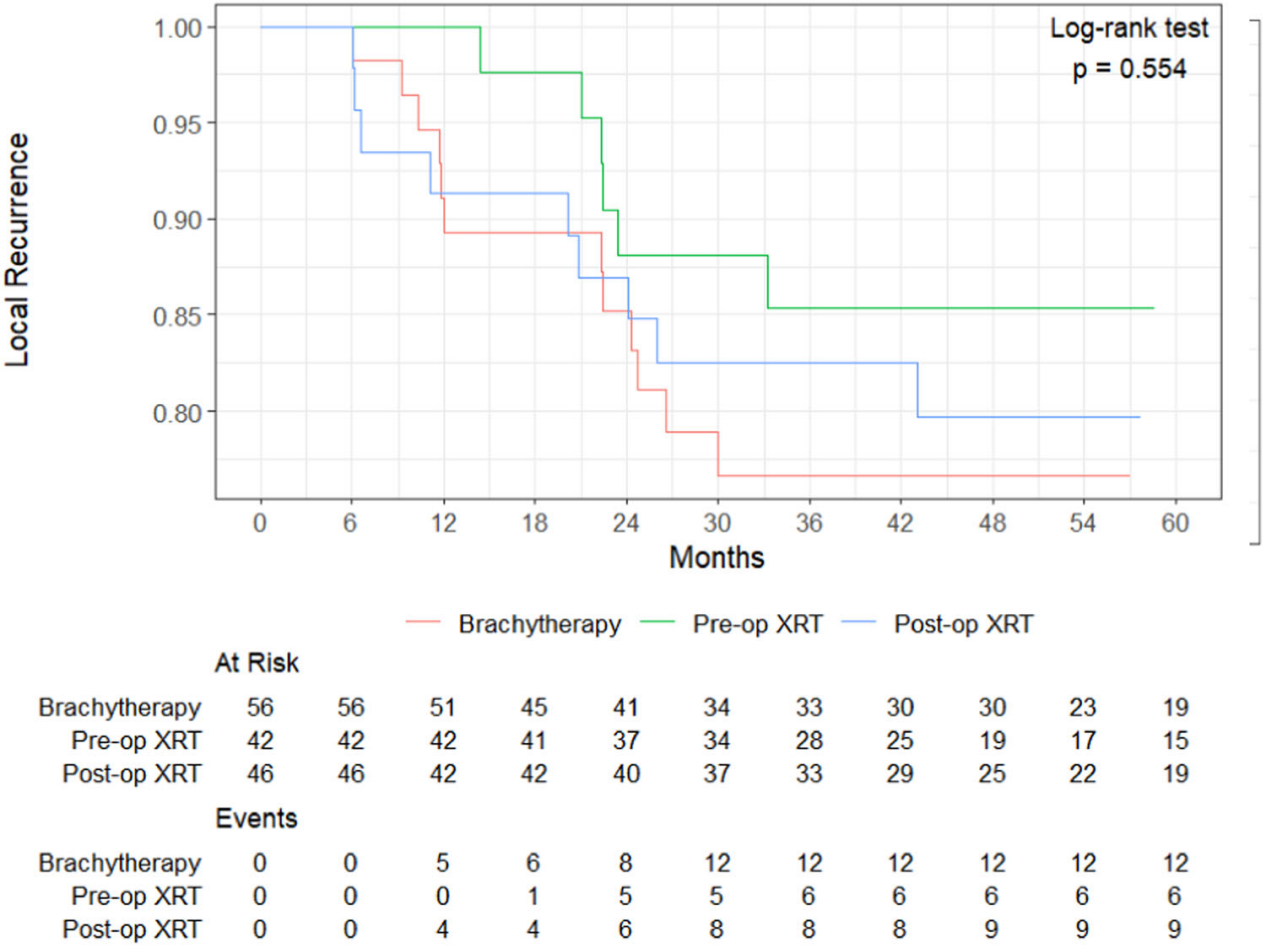
Radiotherapy groups local recurrence Kaplan Meier Curves.

This study aims to assess the clinical and oncologic outcomes of BRT versus both neoadjuvant EBRT (Neo‐EBRT) and adjuvant‐EBRT (AD‐EBRT) in the setting of high‐grade soft tissue sarcoma. The primary outcomes of this study are wound complications and local recurrence. It analyzes the impact of various tumor characteristics on the oncologic outcomes and wound complication rates associated with the radiotherapy modalities being studied. This study will perform a multivariate analysis to control for various factors that may influence the oncologic outcomes and to more accurately assess the impact of brachytherapy on the risk of wound complications and local recurrence (LR) Figure 1.

## 2 Methods

This retrospective cohort study of 144 consecutive patients treated surgically for soft tissue sarcoma at the same institution from 2010 to 2021. All patients treated for a grade 2 or 3 (specify pathology grade‐ FNCLCC [18]) soft tissue sarcoma by a single surgeon (HTT) were included. From all patients who presented with a soft tissue sarcoma, individuals with low‐grade sarcomas or those treated without radiation, patients with metastatic disease at presentation, patients managed nonoperatively, those who had incomplete medical records, or had less than 2 year of postoperative follow‐up were excluded from this study. Retroperitoneal, head and neck tumors or pelvic resections were also excluded. The study was approved by our IRB.

Demographic and clinical data was collected for each patient including patient age, gender, race, ethnicity, body mass index (BMI), presence of diabetes, smoking status, treatment with preoperative or postoperative chemotherapy and radiotherapy in the form of EBRT or BRT. Additionally, tumor characteristics were reviewed by a dedicated musculoskeletal pathologist. Variables included: tumor type, size, grade, location, viability, tumor size, resection margins, and the presence of disease in the final specimen in the context of a previous unplanned resection. Patients with synovial sarcoma, clear‐cell sarcoma, angiosarcoma, rhabdomyosarcoma, and epithelioid sarcoma (SCARE) were identified and specifically assessed, as these types of sarcomas are most likely to metastasize to locoregional lymph nodes [19]. Myxofibrosarcoma (MFS) also separately assessed due to pattern of recurrence [20]. Percent viability of the tumor was grouped by < 5%, 5%–50%, and > 50% consistent with the literature [21]. In the case of large soft tissue defect after the resection, the need for complex soft tissue reconstructive procedures including locoregional or free flaps was documented. Clinical outcomes were recorded by the primary musculoskeletal oncology surgeon including LR, distant metastasis, any complications, local recurrence, metastasis and mortality. Wound complications were reported and stratified. These included surgical site infections (SSI), wound dehiscence, hematoma and seromas. SSIs were defined as an infection related to a surgical procedure that occurs near the surgical site within 30 days following surgery or up to 90 days following surgery if an implant is involved, per the CDC [22]. SSI were further divided into superficial (involving only skin and subcutaneous tissues) and deep (involving deeper softer tissues of the incision), but were grouped together for analysis [22]. Return to OR for debridement or soft tissue reconstructive procedures were noted. Cardiac events and pulmonary embolisms were not included in the reporting for this study.

All patients treated with brachytherapy received care from a single radiation oncologist (MK) with specialization in brachytherapy. Brachytherapy catheters were implanted intraoperatively immediately after surgical‐wide excision and subsequently loaded, on average, 5 days postoperatively, achieving a peripheral tumor dose of 34 Gy. Alternatively, EBRT was given preoperatively or postoperatively with three‐dimensional conformal radiation therapy and more recently with intensity‐modulated radiation therapy, in accordance with previously described techniques; typically 50 Gy and 60 Gy for neoadjuvant and adjuvant settings, respectively [15, 23, 24]. Patients were seen daily during their hospitalization, and those who received brachytherapy were seen twice daily during their outpatient treatments until the catheters were removed.

Descriptive statistics were utilized to compare brachytherapy to other radiation therapies to provide an understanding of their impact on LR and complication rates. Treatment groups were stratified into BRT, Neo‐EBRT, and Ad‐EBRT. Quantitative variables are expressed as mean and standard deviation or median and interquartile range depending on their distribution. Categorical variables are expressed as absolute values or percentages. Comparative analyses, including chi‐square or Fisher's exact tests and t‐tests or Mann‐Whitney U tests, were applied to explore associations and differences in categorical and continuous variables, respectively. Subgroup analyses further stratify the data based on tumor subtype, grade, and patient demographics to investigate potential variations in the impact of different radiation therapies. Multivariate regression, using Cox models and logistic regression, were utilized to identify independent predictors of overall survival and assess factors associated with specific outcomes. Survival analysis techniques, such as Kaplan‐Meier curves, log‐rank tests, and Cox Proportional‐Hazards models, were employed to LR survival rates and assess differences between different radiation treatment groups, while adjusting for relevant covariates. Data were analyzed using STATA version 18.0 (STATA Corp, USA) and R version 3.5.1 (R Foundation for Statistical Computing). Statistical significance was defined as a p value less than 0.05.

A total of 144 patients were enrolled in this study. 56 patients were treated with BRT only and were in group A. 42 patients were treated with Neo‐EBRT group B. 46 patients were treated with Ad‐EBRT, group C. There was no difference in the distribution of patients under 40 years old, between 40 and 60 years old, and greater than 60 years old between treatment groups (*p* = 0.48). There was no difference in the distribution of patients with a BMI less than 30, between 30 and 40, and greater than 40 between treatment groups (*p* = 0.06). The average follow‐up time between groups was similar, with 64.2 months in Group A, 56.2 months in Group B, and 69.2 months in Group C (*p* = 0.20). There was no difference in the distribution of STSs of the upper extremity, pelvic girdle and trunk, and the lower extremity (*p* = 0.27). The prevalence of neoadjuvant chemotherapy in addition to radiation therapy was similar between all groups, with 35.7% (20/56) in group A, 50.0% (21/42) in group B, and 26.1% (12/46) in group C (*p* = 0.07). Fungating masses trended towards significance with 1.8% (1/56) in group A, 14.3% (6/42) in group B, and 8.7% (4/46) in group C (*p* = 0.06). The prevalence of positive margins also trended towards significance, with 8.9% (5/56) in group A, 0.0% (0/42) in group B, and 10.9% (5/46) in group C (*p* = 0.07) (Table 1).

**Table 1:** Brachytherapy clinical and demographic descriptive analysis.

	Total		A (Brachytherapy)		B (Preop)			C (Postop)		
Variable	Mean (std)/*n* (%)		Mean (std)/*n* (%)		Mean (std)/*n* (%)			Mean (std)/*n* (%)		*p* value
Age										0.48
< 40	28	19.4	12	21.4	7	16.7		9	19.6	
40–60	62	43.1	23	41.1	22	52.3		17	36.9	
> 60	54	37.5	21	37.5	13	31.0		20	43.5	
Gender										
M	83	57.6	34	60.7	27	64.3		22	47.8	0.25
F	61	42.4	22	39.3	15	35.7		24	52.2	
BMI										**0.06**
< 30	96	66.7	37	66.1	34	80.9		25	54.3	
30–40	37	25.7	16	28.6	7	16.7		14	30.4	
> 40	11	7.6	3	5.3	1	2.4		7	15.3	
Location										0.27
Upper extremity	36	25.0	12	21.4	10	23.8		14	30.4	
Trunk and pelvic Girdle	19	13.2	4	7.2	8	19.1		7	15.2	
Lower extremity	89	61.8	40	71.4	24	57.1		25	54.4	
Size										0.28
< 5	24	16.7	8	14.2	6	14.3		10	21.7	
5–10	56	38.9	24	42.9	20	47.6		12	26.1	
> 10	64	44.4	24	42.9	16	38.1		24	52.2	
Depth										0.32
Deep	47	32.7	16	28.6	12	28.6		19	33.9	
Superficial	97	67.3	40	71.4	30	71.4		27	66.1	
Neoadj‐Chemo										0.07
Yes	53	36.8	20	35.7	21	50.0		12	26.1	
Tumor viability										0.66
> 50% viable	60	41.7	25	44.6	15	35.7		20	43.5	
6%–50%	73	50.7	27	48.2	25	59.5		21	45.6	
< 5% viable	11	7.6	4	7.2	2	4.8		5	10.9	
Setting										0.57
Unplanned	47	32.6	20	35.7	11	26.2		16	34.8	
Planned	97	67.4	36	64.3	31	73.8		30	65.2	
Fungating										0.06
Yes	11	7.6	1	1.8	6	14.3		4	8.7	
Diabetes										0.15
Yes	30	20.8	9	16.1	7	16.7		14	30.4	
Margins										0.07
Positive	10	6.9	5	8.9	0	0.0		5	10.9	
Complex ST recon										0.94
Yes	15	10.4	6	10.7	5	11.9		4	8.7	
Wound complications										**0.02**
Yes	48	33.3	14	25.0	21		50.0	13	28.3	
SSI										**0.04**
Yes	23	16.0	6	10.7	12		28.6	5	10.9	
Dehiscence										0.50
Yes	19	14.6	5	8.9	7		16.7	7	15.2	
I&D needed										0.32
Yes	24	17.0	8	14.5	13		24.4	6	13.3	
Local recurrence										0.66
Yes	27	18.7	12	21.4	6		14.3	9	19.6	
Follow‐up										
Months	63.47	34.19	64.20	40.40	56.16		20.40	69.24	35.42	0.20

*Note:* 1: One‐way ANOVA. 2: Chi‐squared test. 3: Fisher's exact test.

## 3 Results

When evaluating grouped wound complications, there was a significantly greater incidence of grouped wound complications in Neo‐EBRT with 50% (21/42) compared to both brachytherapy with 25% (14/56) and AD‐EBRT with 28.3% (13/46) (***p*** = **0.02**). When evaluating for surgical site infections only, there was a significantly greater incidence in Neo‐EBRT with 28.6% (12/42) compared to both brachytherapy with 10.7% (6/56) and AD‐EBRT with 10.9% (5/46) (***p*** = **0.04**). There was no difference in the incidence of wound dehiscence with 8.9% (5/56) in group A, 16.7% (7/42) in group B, and 15.2% (7/46) in group C (*p* = 0.50). The number of complex soft tissue reconstructions required was similar between groups (*p* = 0.94). Return to OR for Irrigation & Debridement were similar between groups (*p* = 0.32). LR rates were similar between groups with 21.4% (12/56) in group A, 14.3% (6/42) in group B, and 15.2% (9/46) in group C (*p* = 0.66) Tables 2, 3, 4, 5.

**Table 2:** Wound complications univariate logistic regression.

Variable/reference	OR	95% CI	*p* value
Radiation treatment/Brachytherapy			**0.03**
Preop EBRT	2.999	(1.289; 7.195)	
Postop EBRT	1.182	(0.486; 2.867)	
Age groups/< 40			0.24
40–60	0.549	(0.219; 1.376)	
> 60	0.444	(0.169; 1.149)	
Gender/M			0.12
F	0.567	(0.271; 1.154)	
BMI per group/< 30			0.06
30–40	1.556	(0.690; 3.448)	
> 40	4.472	(1.249; 18.247)	
Location/Upper Extremity			**0.06**
Trunk and pelvic girdle	6.875	(2.021; 25.992)	
Lower extremity	2.672	(1.059; 7.732)	
Tumor size/< 5			0.33
5–10	1.349	(0.491; 3.989)	
> 10	1.186	(0.437; 3.466)	
% Viable tumor per group/> 50% viable			0.43
6%–50%	1.574	(0.761; 3.320)	
< 5% viable	0.949	(0.191; 3.733)	
Depth/Deep			0.90
Superficial	0.954	(0.459; 2.019)	
Neoadjuvant chemotherapy/no			0.22
Yes	1.556	(0.762; 3.172)	
Setting/unplanned			0.53
Planned	1.272	(0.607; 2.752)	
Fungating/no			0.39
Yes	1.744	(0.479; 6.104)	
Diabetes/no			0.67
Yes	1.205	(0.508; 2.760)	
Complex ST recon needed/no			0.26
Yes	1.878	(0.620; 5.580)	

**Table 3:** Local recurrence univariate Cox regression.

**Variable**	**HR**	**95% C.I**	***p* value**
Setting/brachytherapy			
Brachytherapy	1		0.28
Preop EBRT	0.583	(0.219; 1.555)	
Postop EBRT	0.835	(0.352; 1.984)	
Age group			
< 40	1		0.08
40–60	0.549	(0.538; 10.951)	
> 60	0.444	(0.918; 17.776)	
Gender/M			0.27
F	1.531	(0.719; 3.258)	
BMI per group/< 30			0.07
30–40	2.091	(0.949; 4.606)	
> 40	1.357	(0.308; 5.973)	
Location/upper extremity			0.18
Trunk and pelvic girdle	0.209	(0.026; 1.674)	
Lower extremity	0.850	(0.370; 1.956)	
Tumor size/< 5			0.13
5–10	1.921	(0.415; 8.892)	
> 10	3.108	(0.714; 13.521)	
Wound complications/no			0.72
Yes	1.151	(0.527; 2.514)	
% Viable tumor per group/> 50% viable			0.34
6%–50%	1.485	(0.656; 3.361)	
< 5% viable	1.219	(0.263; 5.642)	
Depth/deep			**0.02**
Superficial	3.095	(1.058; 9.056)	
Type of STS/rest			
SCARE	0.423	(0.100; 1.786)	0.24
MFS	1.997	(0.839; 4.754)	0.12
Neoadj‐Chemo/no			0.69
Yes	0.851	(0.382; 1.894)	
Setting/unplanned			< 0.001*
Planned	0.110	(0.044; 0.273)	
Fungating/no			0.02*
Yes	3.248	(1.227; 8.596)	
Diabetes/no			0.34
Yes	1.497	(0.655; 3.421)	
Complex ST recon/no			**0.08**
Yes	2.417	(0.911; 6.408)	
Margins/neg			< 0.001*
Positive	16.227	(8.353; 44.254)	

**Table 4:** Local recurrence multivariate regression model.

Variable		HR	(95% CI)	*p* value
Grade	2	1		0.40
	3	0.671	(0.263; 1.712)	
Tumor size				0.47
	< 5	1		
	5–10	1.373	(0.286; 6.585)	
	> 10	1.750	(0.382; 8.017)	
Margins	No	1		**< 0.0001**
	Yes	19.904	(7.542; 52.528)	
Setting	Brachytherapy	1		0.84
	Preop EBRT	1.118	(0.373; 3.352)	
	Postop EBRT	0.978	(0.395; 2.423)	

*Note:* Harrel's C: 0.689. AIC: 236.62. BIC: 244.39.

**Table 5:** Wound complication logistic multivariate regression model.

Variable		OR	(95% CI)	*p* value
Grade	2	1		0.52
	3	1.353	(0.555; 3.454)	
Tumor size	< 5	1		0.57
	5–10	1.406	(0.450; 4.721)	
	> 10	1.139	(0.370; 3.731)	
Location	Upper extremity	1		**0.003**
	Trunk and pelvic girdle	8.105	(2.139; 34.676)	
	Lower extremity	3.610	(1.310; 11.525)	
Setting	Brachytherapy	1		**0.005**
	Preop EBRT	3.940	(1.538; 10.620)	
	Postop EBRT	1.035	(0.372; 2.816)	
BMI	< 30	1		**0.019**
	30–40	2.625	(1.048; 6.733)	
	> 40	8.654	(2.109; 40.956)	

*Note:* Harrel's C: 0.7684. AIC: 175.29. BIC: 204.99.

Next, a univariate analysis was performed to assess the risk of wound complications for multiple variables. Univariate analysis showed that there was an increased risk of wound complications with Neo‐EBRT (HR = 2.999, 95% CI [1.289, 7.195]) but not AD‐EBRT (HR = 1.182, 95% CI [0.486, 2.867]) when compared to brachytherapy (***p*** = **0.03**). BMI > 40 (HR = 4.472, 95% CI [1.249, 18.247]) approached but did not reach a significantly increased risk of wound complications, and BMI 30–40 (HR = 1.556, 95% CI [0.690, 3.448]) was not associated with an increased risk of wound complications when compared to a BMI < 30 (*p* = 0.06). Tumors of the trunk and pelvic girdle (HR = 6.875, 95% CI [2.021, 25.992]) and lower extremity (HR = 2.672, 95% CI [(1.059, 7.732)]) trended towards an increased risk of wound complications when compared to tumors of the upper extremity (*p* = 0.06). Neoadjuvant chemotherapy (HR = 1.556, 95% CI [0.762, 3.172]), in addition to radiation therapy, was not associated with an increased risk of wound complications (*p* = 0.22).

Then a univariate analysis was performed to assess the risk of LR for multiple variables. Univariate analysis showed that there was no significantly increased risk of LR for Neo‐EBRT (HR = 0.992, 95% CI [0.356, 2.761]) and AD‐EBRT (HR = 0.893, 95% CI [0.369, 2.156]) when compared to brachytherapy (*p* = 0.28). Anatomical location of STS was not significantly associated with LR (*p* = 0.18). Wound complications (HR = 1.287, 95% CI [0.586, 2.822]) were not associated with an increased risk of LR whe compared to no wound complications (*p* = 0.72). Superficial tumors were associated with a significantly increased risk of LR (HR = 3.095, 95% CI [1.058, 9.056]) when compared to deep tumors (*p* = **0.02**). Neoadjuvant chemotherapy in addition to radiation therapy was not associated with a reduced risk of LR (*p* = 0.69). Planned excisions (HR = 0.110, 95% CI [0.044, 0.263]) were associated with a significantly decreased risk of LR (***p*** =** < 0.001**). Fungating masses (HR = 3.248, 95% CI [0.1.227, 8.596] were associated with a significantly increased risk of LR (***p*** = **0.02**). Positive margins (HR = 16.227, 95% CI [8.353, 44.254] were associated with a significantly increased risk for LR (***p*** =** < 0.001**).

Next, a multivariate regression cox analysis was performed to explore the impact of multiple independent associations of several variables with the hazard of LR in this specific context. Positive margins were a predictive factor of LR when controlling for grade, size, and radiation therapy modality. Positive margins were predictive of the event (HR = 19.350 95% CI [7.339, 15.021; ***p*** =** < 0.0001**). When controlling for all other variables, radiation modality did not affect LR.

A multivariate regression analysis was then performed to explore the impact of multiple important associations of several variables with the hazard of grouped wound complications in this specific context. When controlling for grade and size, the anatomical location of STS, radiation treatment modality and BMI were predictive factors. Tumors of the trunk and pelvic girdle (HR = 8.014 95% CI [2.114, 34.316]) and lower extremity (HR = 3.505 95% CI [1.271; 11.186]) were in fact predictive of an event when compared to tumors of the upper extremity (***p*** = **0.02**). Additionally, Neo‐EBRT (HR = 4.028 95% CI [1.483, 11.590]) was predictive of the event while AD‐EBRT (HR = 1.160 95% CI [0.396, 3.338]) was not predictive of the event when compared to brachytherapy (***p*** = **0.007**). BMI > 40 (HR = 7.907 95% CI [1.817, 39.934]) was predictive of the event while BMI 30‐40 (HR = 2.516 95% CI [0.978, 6.610]) was not predictive of the event when compared to BMI < 30 (***p*** = **0.006**). This indicates that Neo‐EBRT, tumor location and BMI > 40 are associated with a significantly increased risk of grouped wound complications when controlling for all other variables.

## 4 Discussion

The purpose of this study is to assess the clinical and oncologic outcomes of BRT versus both Neo‐EBRT and AD‐EBRT in the setting of high‐grade soft tissue sarcomas. The primary outcomes of this study are wound complications and local recurrence. This study found that BRT is an excellent radiation treatment modality. There was no significant difference in LR between BRT and EBRT, while controlling for other important variables, indicating a comparable local control. This study also found that the incidence of grouped wound complications was lower in brachytherapy when compared to Neo‐EBRT but similar to AD‐EBRT. Specifically, the prevalence of SSI was lower in brachytherapy than in Neo‐EBRT. This study includes a large number of patients treated with brachytherapy, that is demographically similar with an average patient follow up greater than 5 years.

Brachytherapy may improve upon the shortcomings of EBRT without increasing risk for tumor recurrence. There was no difference in size distribution between treatment groups, ensuring that brachytherapy was not favored for smaller tumors. This study found that there was no difference in the need for complex soft tissue reconstruction between brachytherapy and EBRT. Radiation therapy can cause significant soft tissue damage through reactive oxygen species formation and tissue ischemia, which puts patients at risk of extensive soft tissue complications, sometimes requiring reoperation [25]. Despite this, brachytherapy delivers a very high dose to a much smaller area and can, therefore, spare more normal tissue than EBRT. The incidence of grouped wound complications was 25% in the brachytherapy group. This is similar to multiple other studies that showed wound complication rates ranged from 5% to 75% in patients treated with brachytherapy [2, 26, 27]. These rates are lower than both Neo‐EBRT but similar to AD‐EBRT which were 50% and 28%, respectively. This study's EBRT wound complication rates are similar to other reported studies with complication rates of Neo‐EBRT of 40% and AD‐EBRT around 20% [28, 29]. The LR rate was 21.4% in the brachytherapy cohort, which was comparable to both Neo‐EBRT, with 14.3%, and AD‐EBRT, with 19.6%, with an average of 5 years of follow‐up. Studies show that 5‐year LR rates with brachytherapy, Neo‐EBRT, and AD‐EBRT are similar but range from roughly 6% to 30% [24, 30, 31]. The referral pattern at this institution explains an unfortunately high number of unplanned excisions which may bias the surgical complexity, final surgical margins, wound complications, and LR [32].

Radiation modality is an essential factor when considering peri‐operative wound complications in the treatment of STS. Based on univariate analysis, this study found that there were roughly a three times increased risk of experiencing grouped wound complications when using Neo‐EBRT compared to brachytherapy. Similarly, there was a decreased risk when comparing AD‐EBRT to neo AD‐EBRT, compatible with known literature [29, 33]. Current literature shows diverging results when comparing brachytherapy to EBRT for wound complications. Some studies note an increase in perioperative wound complications in brachytherapy compared to traditional EBRT, specifically, chronic edema, infection, and radiation dermatitis [11, 15]. These studies hypothesize that their results may be due to physician technique or high variability in the definition of a complication [34]. Other studies note a non‐inferior risk of wound complications in brachytherapy compared to traditional EBRT [35, 36]. After controlling for other variables that could bias the analysis, the multivariate analysis in this study continued to show that brachytherapy results in a lower risk of wound complications than Neo‐EBRT. This study highlights that brachytherapy may be more favorable than traditional EBRT when considering wound complications. Further analysis and classification of radiation‐associated surgical site complications in STS should be established for large‐scale studies. Differentiating superficial from deep SSI, aseptic dehiscence, seroma, hematoma and return to the OR is essential to study to mitigate those adverse events appropriately.

Another important consideration is presence of obesity for complication risk. Previous studies have reported that obesity increases the risk of wound complications and this analysis similarly shows that people with severe obesity trend towards higher risk of wound complications [37]. When controlling for other factors, obesity proved to be a strong predictive factor for increased risk of wound complications. This study found that trunk, pelvic girdle, and lower extremity tumors are associated with a greater risk of wound complications on both univariate and multivariate analysis. Both anecdotally described and in comparison to the results of other studies, these findings are supported by studies illustrating that tumors of the lower extremity, specifically the proximal medial thigh, are associated with an increased risk for wound complications [17, 34, 38]. These high‐risk areas deserve particular attention or intervention including wound VAC therapy, lymphatics ligation techniques, hemostatic agents, etc. When controlling for other variables, larger size and chemotherapy use were not associated with an increased risk of wound complications in this study.

Local control appears to be comparable between brachytherapy and EBRT. Univariate and multivariate analyses both highlight similar risk of local recurrence indicating that local control may not be a limitation for brachytherapy use when deciding between treatment modalities. This finding is similar to multiple other studies that describe no difference in the risk of LR between radiation modalities [2, 9, 15]. Additionally, multivariate analysis showed that grade and size were not predictive factors for LR. This study also found that positive margins conferred a nearly 16 times increased risk of LR on univariate analysis. When controlling for other factors on the multivariate analysis, there was a nearly 19 times increased risk of LR. These findings are similar to other studies that also found that positive margins increase risk of LR and highlight the importance of R0 margins [39, 40, 41]. Unplanned excisions were associated with an increased risk of LR in this study and influenced surgical site contamination and final margins. This observation has been published [32]. This finding is similar to other studies and highlights the importance of compliance to preoperative workup and continuous efforts to increase awareness of STSs to other providers [32, 42]. This study found that superficial tumors were associated with an increased risk of LR in concert with other studies that have found that tumor depth may not be associated with LR [43, 44]. These results may be explained by the fact that superficial tumors are more likely to be associated with unplanned excisions associated with increased risk for LR [45]. A multivariate analysis dedicated to unplanned excision is warranted.

Given the findings in this study, brachytherapy may be a beneficial treatment modality that can be used for STSs based on specific criteria. An algorithmic approach to determine its best applications may help clinicians. This study proposes that brachytherapy should be used when neoadjuvant tumor size reduction will not change surgical resection. Brachytherapy can be even more beneficial in areas like the pelvic girdle, and proximal thigh that are at risk for wound complications, particularly in obese patients. Based on the current analysis, size should not be a limiting factor, although careful technical planning with a brachytherapist is warranted for lesion larger than 10‐15 cm. Neo‐EBRT should be used when a large tumor is in close proximity to neurovascular structures or other critical anatomical structures, and there is a benefit to reducing the tumor size before surgery. Neo‐EBRT can be used in conjunction with chemotherapy to achieve additional tumor shrinkage. In these cases, an additional BRT boost can still be performed to address close margins. Neo‐EBRT can also be used if there is a need to treat larger soft tissue volumes after unplanned excision. AD‐EBRT may be used for fungating tumors or a medical indication requiring immediate tumor resection or when brachytherapy is not available at the institution. A previous study determined that an algorithmic approach in which brachytherapy was proposed for patients with an anticipated close margin, a positive surgical margin, and for patients who are unlikely to receive a complex soft tissue procedure [11]. The algorithm aims to supplement this previous study and help clinitions decide what the best radiation modality to use is for each clinical scenario.

This study is not without limitations. First, this study was performed at a single institution which may lead towards bias in patient population. Next, there are inherent shortfalls of retrospective studies that have been previously described [46]. Additionally, this study did not examine the impact of radiation modalities on specific STS types. It has been previously described that different histological subtypes may respond differently to radiation therapy [47]. This study also did not examine different radiation techniques on these outcomes, although all radiation treatments were uniformly performed at this institution. Additionally, this study recognizes that the generalizability of this study may be limited given the variability in catheter placement technique and varying tumor characteristics. Although brachytherapy may improve patient satisfaction given its short course, multiple physicians and team members are required to make the impact and experience of any treatment modality beneficial to patients [48].

For future studies, collaborative effects are necessary to better understand the effects of radiotherapy and clinical outcomes in patients with soft tissue sarcomas. Although this study's multivariate analysis was adequate for wound complications and local recurrence, it was not strong enough for an analysis on metastasis and disease‐specific survival. Collaborative databases would allow for stronger analyses on these oncologic outcomes. Additionally, studies need to isolate specific treatment modalities with specific tumor characteristics and in specific settings to optimize therapy and patient selections. Further analysis and classification of radiation‐associated surgical site complications in STS should be established for larger scale studies Differentiating superficial from deep SSI, aseptic dehiscence, seroma, hematoma and return to the OR is essential to appropriately study interventions that will treat those adverse events.

## 5 Conclusion

Brachytherapy is a valuable treatment modality that offers clinical advantages when compared to Neo‐EBRT in soft tissue sarcomas. This study found that, while condensing radiation treatment to less than 1 week postoperatively, BRT offers a nearly 3 times lower risk of wound complications and a comparable local control. Based on the findings in this study, this study proposes an algorithm in which brachytherapy should be used for tumors of any size where neoadjuvant tumor size reduction will not significantly change the surgical resection functional deficit.

## Ethics Statement

Ethical approval was obtained through the University of Miami IRB 20230560 for this Study.

## Conflicts of Interest

The authors declare no conflicts of interest.

## Synopsis

Brachytherapy is a valuable treatment modality that offers clinical and logistical advantages when compared to the conventional Neo‐EBRT in soft tissue sarcomas. Brachytherapy offers a lower risk of wound complications and a comparable local control. This manuscript presents decision‐making strategies for determining the appropriate radiation modality for specific circumstances.

## Data Availability

The data that support the findings of this study are available from the corresponding author upon reasonable request.
